# Anti-Alpha-Amino-3-Hydroxy-5-Methyl-4-Isoxazolepropionic Acid Receptor Encephalitis: A Review

**DOI:** 10.3389/fimmu.2021.652820

**Published:** 2021-05-21

**Authors:** Tian-Yi Zhang, Meng-Ting Cai, Yang Zheng, Qi-Lun Lai, Chun-Hong Shen, Song Qiao, Yin-Xi Zhang

**Affiliations:** ^1^ Department of Neurology, Tongde Hospital of Zhejiang Province, Hangzhou, China; ^2^ Department of Neurology, Second Affiliated Hospital School of Medicine Zhejiang University, Hangzhou, China; ^3^ Department of Neurology, Zhejiang Hospital, Hangzhou, China

**Keywords:** alpha-amino-3-hydroxy-5-methyl-4-isoxazolepropionic acid receptor, neuronal surface antibody, autoimmune encephalitis, limbic encephalitis, immunotherapy

## Abstract

Anti-alpha-amino-3-hydroxy-5-methyl-4-isoxazolepropionic acid receptor (AMPAR) encephalitis, a rare subtype of autoimmune encephalitis, was first reported by Lai et al. The AMPAR antibodies target against extracellular epitopes of the GluA1 or GluA2 subunits of the receptor. AMPARs are expressed throughout the central nervous system, especially in the hippocampus and other limbic regions. Anti-AMPAR encephalitis was more common in middle-aged women and most patients had an acute or subacute onset. Limbic encephalitis, a classic syndrome of anti-AMPAR encephalitis, was clinically characterized by a subacute disturbance of short-term memory loss, confusion, abnormal behavior and seizure. Magnetic resonance imaging often showed T2/fluid-attenuated inversion-recovery hyperintensities in the bilateral medial temporal lobe. For suspected patients, paired serum and cerebrospinal fluid (CSF) testing with cell-based assay were recommended. CSF specimen was preferred given its higher sensitivity. Most patients with anti-AMPAR encephalitis were complicated with tumors, such as thymoma, small cell lung cancer, breast cancer, and ovarian cancer. First-line treatments included high-dose steroids, intravenous immunoglobulin and plasma exchange. Second-line treatments, including rituximab and cyclophosphamide, can be initiated in patients who were non-reactive to first-line treatment. Most patients with anti-AMPAR encephalitis showed a partial neurologic response to immunotherapy.

## Introduction

Encephalitis is an infectious or inflammatory disorder of the brain parenchyma (1). There were 5-10 per 100,000 inhabitants suffering from encephalitis every year across all age groups (2, 3). Though classically attributed to infection, an autoimmune basis was reported with similar frequency for encephalitis (4). Autoimmune encephalitis (AE) is the umbrella term for autoimmune disorders in the central nervous system (CNS) characterized by the presence of autoantibodies against intracellular or membrane antigens (5). Over the past decade, the identification of an increasing number of antibodies have aided in the identification and characterization of AE (4). Anti-alpha-amino-3-hydroxy-5-methyl-4-isoxazolepropionic acid receptor (AMPAR) encephalitis, a rare subtype of AE mediated by AMPAR antibodies, was first reported by Lai et al. in 2009 (6). More than half of the patients were characterized by limbic encephalitis (LE), including short-term memory loss, confusion, abnormal behavior and seizures. In recent years, anti-AMPAR encephalitis has been increasingly reported with atypical clinical manifestations.

We searched PubMed, Web of Science and Embase for all articles published in English between April 2009 and November 2020, using the search terms [(AMPA OR AMPAR OR anti-AMPA OR anti-AMPAR OR AMPAR-antibody OR AMPA receptor OR anti-alpha-amino-3-hydroxy-5-methyl-4-isoxazolepropionic acid) AND (encephalitis OR autoimmune encephalitis OR limbic encephalitis)]. We identified 66 cases from 6 case series and 20 individual case reports (6–31) (**Table 1** and **Supplementary Figure 1**). We summarized the clinical presentations, diagnostic tests and treatments of anti-AMPAR encephalitis, in order to raise the awareness among neurologists (**Supplementary Table 1**).

**Table 1:** Summary of articles in the review (6–31).

Articles	Country	Number of cases	Sex	Age or age range (years)
Lai et al. (6)	America	10	9/1 (F/M)	38-87
Bataller et al. (7)	Spain	1	F	67
Graus et al. (8)	Spain	2	2 (F)	58, 60
Wei et al. (9)	China	1	F	30
Spatola et al. (10)	Switzerland	1	F	33
Joubert et al. (11)	France	7	4/3 (F/M)	21-92
Li et al. (12)	China	1	F	47
Höftberger et al. (13)	America	22	14/8 (F/M)	23-81
Elamin et al. (14)	Ireland	1	F	73
Dogan Onugoren et al. (15)	Germany	3	1/2 (F/M)	61-62
Quaranta et al. (16)	Italy	1	F	14
Boangher et al. (17)	Belgium	1	F	66
Yang et al. (18)	China	1	M	40
Zhu et al. (19)	China	1	F	54
Koh et al. (20)	Australia	1	M	19
Omi et al. (21)	Japan	1	F	34
Zhu et al. (22)	China	1	M	51
Samad and Wong (23)	Australia	1	F	69
Laurido-Soto et al. (24)	America	2	2 (M)	18, 44
Urriola et al. (25)	Australia	1	F	44
Daneshmand et al. (26)	America	1	F	61
Luo et al. (27)	China	1	F	50
Jia et al. (28)	China	1	M	26
Wei et al. (29)	China	1	F	66
Safadi et al. (30)	America	1	M	30
Qiao et al. (31)	China	1	M	32-month
Total patients		66	44/22 (F/M)	32-month to 92

F, female; M, male.

## Etiology and Pathogenesis

Unlike anti-N-methyl-D-aspartate receptor (NMDAR) encephalitis, only a few patients reported prodromal viral infections (6/66). Forty patients (60.6%) had a history of tumor or detected tumors, with thymoma being the most common (16/40, most of them under 60 years old), followed by small cell lung cancer (SCLC) (8/40), breast cancer (6/40) and ovarian cancer (3/40). There were also cases reporting concomitant medullary thyroid carcinoma, bladder carcinoma, melanoma and Ewing’s sarcoma.

AMPARs are ionotropic receptors which belong to glutamate receptors. AMPARs are mainly heterotetramers and are composed of four subunits (GluA1-4) with several auxiliary subunits (32, 33). AMPARs, together with other ionotropic glutamate receptors, mediate the majority of excitatory synaptic transmission (32, 34). AMPARs, especially GluA1 and GluA2 subunits, are ubiquitously expressed throughout the central nervous system. In particular, there is a rich expression of GluA1/2 and GluA2/3 levels in the hippocampus and other limbic regions (35). The majority of synaptic AMPARs are GluA1/2 subunits and, to a less extent, GluA2/3 subunits in hippocampus (32, 33). GluA1/2 subunits are also widely expressed in the cerebellum, basal ganglia and cerebral cortex (35). The AMPAR antibodies target against the extracellular portion of cell surface proteins, i.e. the extracellular epitopes of the GluA1 or GluA2 subunits of the receptor (36). In vitro experimental studies indicated that AMPAR antibodies selectively decreased the surface synaptic AMPAR clusters and disrupted the balance between internalization and reinsertion of AMPARs, leading to the accumulation of internalized AMPARs (37). The AMPAR-mediated decrease in synaptic transmission led to a compensatory decrease of inhibitory synaptic transmission and an increase of intrinsic excitability (33, 37). Synaptic and neuronal changes induced by antibodies may contribute to the short-term memory loss and seizures in anti-AMPAR encephalitis patients.

## Clinical Manifestations

Most patients (46/66) had an acute or subacute onset. The median age of onset was 57 years old with a wide range from 32-month-old to 92-year-old. The vast majority of patients developed the disease in adulthood (64/66) and patients aged 50 to 70 years old account for nearly 50% among adults. Anti-AMPAR encephalitis was more common in women, with a male to female ratio of about 1:2. The clinical manifestations of encephalitis were varied (**Table 2**).

**Table 2:** Summary of presenting symptoms.

Symptoms	Total, n = 66
**Cognitive impairment**	**81.8% (54/66)**
Short-term memory loss	80.3% (53/66)
Disorientation	19.7% (13/66)
Execution	13.6% (9/66)
Acalculia	1.5% (1/66)
Apraxia	1.5% (1/66)
**Psychiatric disorder**	**80.3% (53/66)**
Abnormal behavior	42.4% (28/66)
Agitation	22.7% (15/66)
Mood disorders	21.2% (14/66)
Psychosis	16.7% (11/66)
Hallucinations	12.1% (8/66)
Delusions	6.1% (4/66)
Confabulation	4.5% (3/66)
**Altered state of consciousness**	**77.3% (51/66)**
Confusion	68.2% (45/66)
Somnolence	7.6% (5/66)
Coma	6.1% (4/66)
**Dyskinesia**	**37.9% (25/66)**
Gait disturbance/ataxia	24.2% (16/66)
Hypermyotonia	9.1% (6/66)
Tremor	6.1% (4/66)
Involuntary movement	4.5% (3/66)
**Seizure**	**28.8% (19/66)**
Status epilepticus	7.6% (5/66)
**Speech disorder**	**15.2% (10/66)**
Aphasia	12.1% (8/66)
Dysfluency	3.0% (2/66)
**Insomnia**	**10.6% (7/66)**
**Autonomic dysfunction**	**9.1% (6/66)**
**Dysarthria**	**4.5% (3/66)**

The non-bold values were the detailed version of the bold values.

Cognitive dysfunction was the most common (54/66) clinical manifestation of anti-AMPAR encephalitis, including short-term memory loss (53/66), disorientation (13/66), executive dysfunction (9/66), etc. It could progress to dementia in severe cases. Psychiatric symptom was the second most common clinical manifestation (53/66), among which abnormal behaviors were most frequent (28/66). Less frequently, manifestations in order of decreasing frequency included agitation (15/66), mood disorders (14/66) and psychosis (11/66). There were 3 patients who developed mutism. An altered level of consciousness was not uncommon as well (51/66). Confusion could develop in most patients (45/66). Patients with dyskinesia (25/66) could manifest as gait disturbance or ataxia, parkinsonism and involuntary movement. Nineteen patients had seizures during the disease course, with only 5 patients having status epilepticus. There were various types of seizures, including myoclonic seizures, paroxysmal feeling of tightness, etc. Speech disorder occurred in 10 patients, mainly manifested as aphasia. In addition, some patients may have insomnia (7/66), autonomic dysfunction (6/66) and dysarthria (3/66).

## Auxiliary Examination

### Magnetic Resonance Imaging

**Figure 1:**
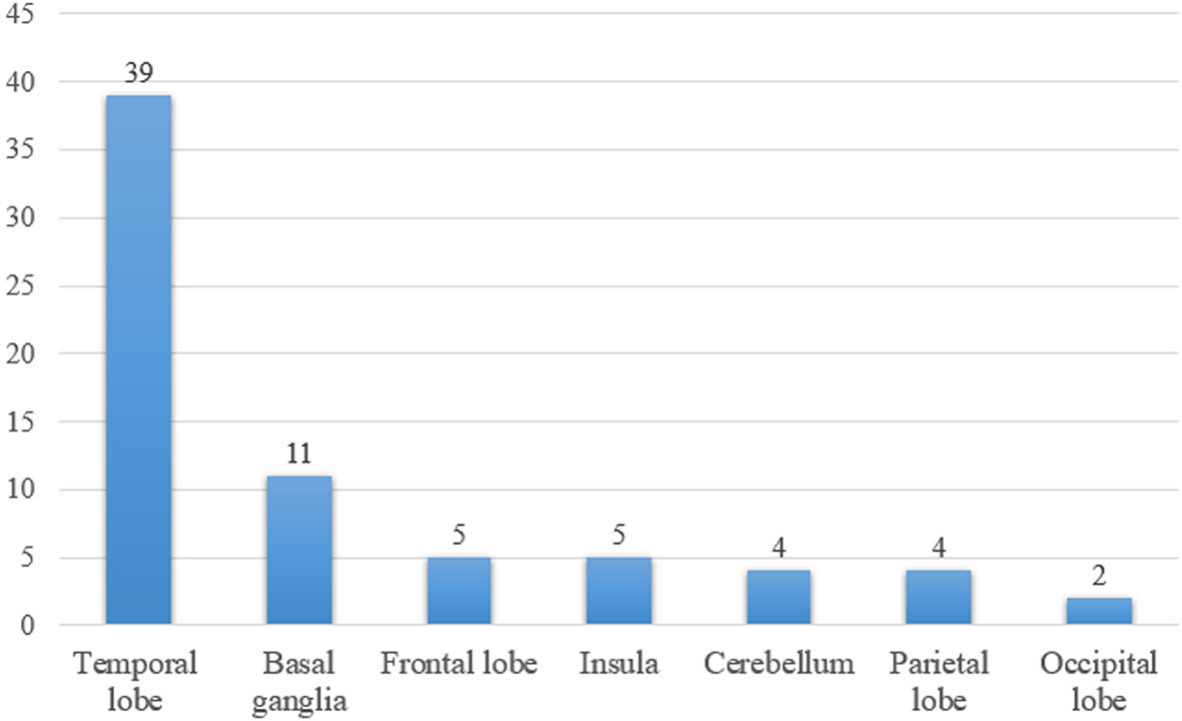
The number of patients with T2/fluid-attenuated inversion-recovery hyperintensity lesions in different brain areas.

**Figure 2:**
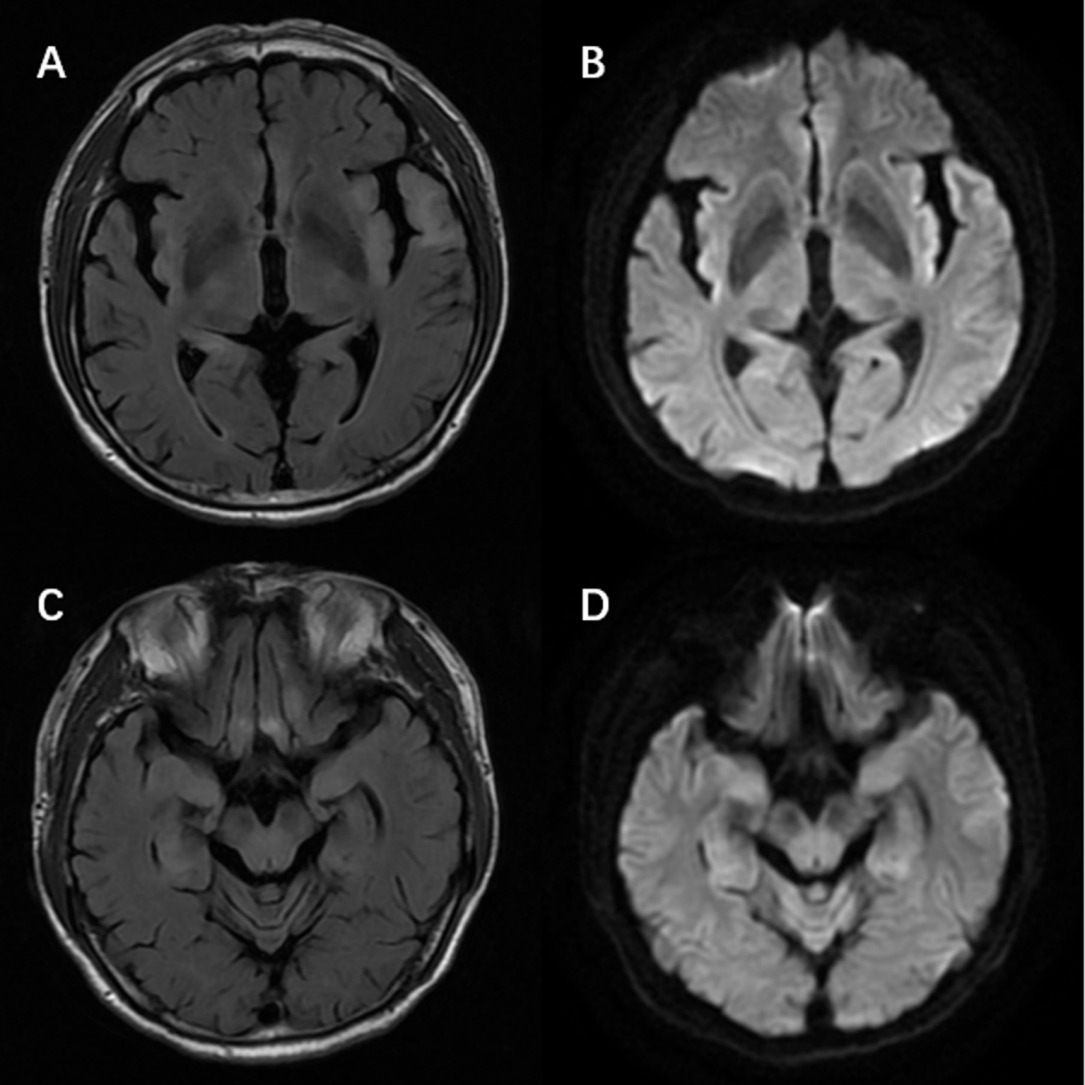
Magnetic resonance imaging of a patient with anti-alpha-amino-3-hydroxy-5-methyl-4-isoxazolepropionic acid receptor encephalitis showed T2/fluid-attenuated inversion-recovery sequency hyperintensity in left temporal lobe, bilateral hippocampus, frontal lobes, insula **(A, C)** with restricted diffusion on diffusion weight imaging at the corresponding region **(B, D)**.

There were 65 patients who underwent magnetic resonance imaging (MRI) scan, with 49 patients showing abnormalities (**Figure 1**). Among them, 39 patients showed increased T2/fluid-attenuated inversion-recovery (FLAIR) sequency signal in the temporal lobe, and the medial temporal lobe was involved in 21 patients (**Figure 2**). Bilateral temporal lobe involvement was more frequent (17 bilateral and 10 unilateral). There were also 11 patients with T2/FLAIR hyperintensity in the basal ganglia, which mainly involved caudate, followed by corpus striatum, putamen. In addition, insula, frontal lobe, cerebellum, parietal lobe, cingulum and occipital lobe were also affected. Leptomeningeal enhancement in the temporal-parietal regions (18) and mild transient contrast enhancement in the hippocampus (6) were reported. In the long term, hippocampal (10, 15) or cortical (9, 25) atrophy could also be seen.

### ^18^F-fluorodeoxyglucose Positron Emission Tomography

There were only 8 cases describing the manifestation of ^18^F-fluorodeoxyglucose positron emission tomography (^18^FDG-PET). Four showed increased metabolism at the onset of disease (cerebellar, medial temporal lobe, hippocampus, striatum, parietal lobe and occipital lobe, basal ganglia) (10, 11, 24), 2 showed normal metabolism (though 1 patient had global hypometabolism on PET 3.5 weeks later) (8, 24), and 2 showed decreased metabolism (caudate, frontal, temporal, occipital and parietal areas) (9, 29). However, the regions with abnormal MRI signals did not match perfectly with those with abnormal ^18^FDG-PET metabolism, mainly because those 2 imaging modalities had a different emphasis on metabolism and structure. The inconsistency might be also due to the different time points when MRI and PET examinations were conducted along the disease course (38). Previous ^18^FDG-PET/MRI studies of AE patients (mainly anti-NMDAR encephalitis) indicated a higher sensitivity of PET than MRI in AE (39, 40). Given the limited sample size, we were unable to determine the sensitivity of PET and MRI for diagnosis of anti-AMPAR encephalitis.

### Electroencephalogram

Among the 53 patients with reported electroencephalogram (EEG) at disease onset, 20 had normal EEG findings. There were 15 patients with epileptiform discharges, such as sharp waves and spike waves. And in 19 patients, EEG showed generalized or focal slowing. A few (4 cases) had epileptiform discharges and slow waves at the same time. However, there were 2 cases with epileptiform waves but no seizures.

### Blood Tests

Routine blood tests showed no obvious abnormalities. Hyponatremia was present in 7 patients. Patients may also have other autoimmune antibodies, such as acetylcholine receptor antibody, thyroid peroxidase antibody and thyroglobulin antibody, autoimmune hepatitis antibodies, anti-nuclear antibody, cardiolipin antibodies, etc.

### Cerebrospinal Fluid

Results of CSF analysis were available for 64 patients, including white blood cells (WBC) in 63 patients and protein levels in 59 patients. More than half of patients (38 cases) had an elevated WBC, with a maximum of 220 cells/μL, mainly composed of lymphocytes. CSF protein was abnormal in 27 patients, with a maximum of 425 mg/dL. Oligoclonal bands were detected in 5 patients.

### Antibody Detection

The positive serum and/or CSF antibody against AMPAR could be used as a reference for the diagnosis of this disease. Cell-based assay (CBA) was recommended due to its high sensitivity and specificity (2). The exact threshold of antibody level for anti-AMPAR encephalitis diagnosis remained inconclusive. Paired serum and CSF samples were available from 43 patients, where 36 had seropositivity and 41 had positive antibodies in the CSF (**Table 3**). In general, CSF AMPAR antibody examination had a higher sensitivity. Considering the distribution of AMPAR and intrathecal synthesis of antibodies (6), the specificity of CSF antibodies was relatively high. Therefore, paired testing of both the serum and CSF samples was recommended, of which the CSF was preferred (41). In cases with matched serum and CSF tests, the titers of AMPAR antibody in serum were higher than that in CSF (15, 27). Notably, patients with only a low-titer of serum antibody should be diagnosed with caution. It had been reported that the AMPAR antibody titer in the CSF gradually decreased after immunosuppressive treatment, as the clinical symptoms relieved (10). However, the association between antibody titer and disease severity remained unknown.

**Table 3:** Summary of antibodies.

Sample types	Total, n = 64/66
Serum+/CSF NA	12.5% (8/64)
Serum NA/CSF +	20.3% (13/64)
Serum+/CSF +	79.1% (34/43^*^)
Serum -/CSF +	16.3% (7/43^*^)
Serum+/CSF -	4.7% (2/43^*^)
GluA1 only	18.8% (9/48^#^)
GluA2 only	58.3% (28/48^#^)
GluA1/2	22.9% (11/48^#^)

Two patients had antibodies with unknown origin (serum or CSF).

^*^Paired samples were available from 43 patients.

^#^There were 48 cases describing exact subunits of alpha-amino-3-hydroxy-5-methyl-4-isoxazolepropionic acid receptor, in which antibodies targeting GluA2 subunit were more common among the reported cases.

CSF, cerebrospinal fluid; NA, not available.

Antibodies against GluA1 and GluA2 subunits, the 2 main subunits of AMPAR, could be detected simultaneously or separately. GluA2-specific antibodies were more commonly reported (**Table 3**). There were no significant differences among clinical presentations between the two subtypes (13).

Nineteen patients had overlapping neural antibodies. Those patients usually had a worse prognosis. The most common concomitant antibody was collapsin response-mediator protein-5 (CRMP5) antibody (6 cases), followed by NMDAR antibody (4 cases), glutamic acid decarboxylase (GAD) antibody (3 cases), voltage-gated potassium channels (VGKC) antibody (3 cases), Sry-like high mobility group box (SOX1) antibody (3 cases), gamma-aminobutyric acid receptor (GABAR) antibody (1 case), antinuclear neuronal antibody type 1 (Hu) antibody (1 case), leucine-rich glioma-inactivated 1 (LGI1) antibody (1 case), amphiphysin antibody (1 case) and voltage-gated calcium channels (VGCC) antibody (1 case).

### Pathology

Unfortunately, there was still no report on the pathology of brain tissue in anti-AMPAR encephalitis. A few cases reported pathological findings coming from concomitant neoplasms that GluA1/2 subunits present in patients’ tumor tissues, which correlated with the patients’ antibody specificity. This indicated that some types of tumors might play a role in triggering this autoimmune disorder (6).

## Diagnosis and Differential Diagnosis

The diagnosis of anti-AMPAR encephalitis was based on the criteria published in the Lancet by Graus et al. (2). In particular, anti-AMPAR encephalitis should be considered in patients with the following characteristics: 1) acute or subacute onset, mostly manifested as short-term memory loss, confusion, abnormal behavior, dyskinesia and seizure; 2) MRI may present with unilateral or bilateral limbic lobe T2/FLAIR hyperintensity; 3) reactive to immunotherapy. In addition, for patients suspected with anti-AMPA encephalitis, a thorough evaluation for tumors, such as thymoma and SCLC, should be conducted.

Diseases mimicking anti-AMPAR encephalitis abound, including infectious, neoplastic, and other immunological diseases (**Table 4**) (2, 42–44). Caution is needed to identify anti-AMPAR-encephalitis from other types of encephalitis, like anti-NMDAR encephalitis and viral encephalitis (**Table 5**) (43, 45, 46).

**Table 4:** Differential diagnosis of anti-AMPAR encephalitis (2, 42–44).

Infectious	Encephalitis caused by various pathogens (e.g. Virus, bacterium, spirochetes, fungus, tuberculosis bacterium, etc.), Creutzfeldt-Jakob disease, Whipple disease
Neurodegenerative	Alzheimer disease, frontotemporal dementia, Lewy body dementia, vascular cognitive impairment
Neoplastic	Primary or secondary central nervous system lymphoma, lymphomatoid granulomatosis, diffuse glioma
Endocrine	Hashimoto encephalopathy
Hereditary	Mitochondrial encephalopathy
Toxic	Substance abuse, carbon monoxide, Wernicke encephalopathy, neuroleptic malignant syndrome
Vascular	Primary central nervous system vasculitis, Behcet disease, Susac syndrome (autoimmune vasculopathy)
Demyelinating	Multiple sclerosis, neuromyelitis optic spectrum disease, acute disseminated encephalomyelitis, myelin oligodendrocyte glycoprotein antibody-associated disease, autoimmune glial fibrillary acidic protein astrocytopathy
Inflammatory	Neurosarcoidosis, Sjogren’s syndrome, systemic lupus erythematosus
Psychiatric	Schizophrenia, bipolar disorder, conversion disorder

AMPAR, alpha-amino-3-hydroxy-5-methyl-4-isoxazolepropionic acid receptor.

**Table 5:** General features distinguishing anti-AMPAR encephalitis from important differential diagnoses (43, 45, 46).

	Anti-AMPAR encephalitis	Anti-NMDAR encephalitis	Viral encephalitis
Age and gender	Middle-aged woman	Especially in girls/young women and children	Onset at any age, no obvious gender difference
Form of onset	Acute or subacute onset, almost no history of pre-infection	Acute or subacute onset, may have a history of pre-infection, such as nausea, vomiting, fever, headache and fatigue, etc.	Most of them are acute onset, the average incubation period of primary infection was 6 days, which can manifest as fever, general malaise, headache, gastrointestinal symptoms, rash, etc.
Main Presenting Symptoms	Short-term memory loss, psychiatric disorder and confusion	Psychosis, language dysfunction, autonomic instability, epileptic seizures and abnormal movements	Psychosis, impaired consciousness, confusion, aphasia, hallucinations, and movement disorder
MRI	24.6% normal, with T2/FLAIR hyperintensity in temporal lobe, basal ganglia insular lobe and other brain areas, mostly bilateral involvement, with brain atrophy in later stage	67% normal or nonspecific changes	T2/FLAIR hyperintensity in the medial temporal lobe, orbital frontal lobe, insular cortex and cingulate gyrus, focal edema, bilateral asymmetry
CSF	More than half of patients had pleocytosis. 45.8% patients had elevated protein. OB can be detected.	About 20% of patients had normal CSF. Some patients may have mildly elevated CSF cells and proteins. OB can be detected.	White blood cells can be normal or slightly elevated, more in 50-100, lymphocytes increased mainly. Protein can be normal or slightly or moderately elevated.
EEG	37.7% patients had normal EEG. 28.3% patients’ EEG may have epileptiform discharges. 35.8% patients’ EEG had general or focal slowing waves.	Patients’ EEG may show delta slowing, dysrhythmias, partial epileptic activity/beta-delta complexes, and also had special manifestations of “extreme delta brush”	Diffuse high amplitude slow waves were common in EEG, especially in unilateral or bilateral temporal and frontal regions. There can even be sharp waves and spikes in the temporal region.
Treatment	Immunotherapy, treatment of tumor and symptomatic treatment	Immunotherapy, treatment of tumor and symptomatic treatment	Antiviral therapy, immunotherapy and symptomatic treatment
Prognosis	Half of the patients left mild cognitive impairment, mental disorders.	Most of the cases can be fully recovered. Some patients recover slowly or incompletely. A small number of patients left mental or movement disorders.	Most patients can be cured after early antiviral treatment. About 10% of patients had sequelae such as paralysis and cognitive impairment.

AMPAR, alpha-amino-3-hydroxy-5-methyl-4-isoxazolepropionic acid receptor; CSF, cerebrospinal fluid; EEG, electroencephalogram; FLAIR, fluid-attenuated inversion-recovery sequency; MRI, magnetic resonance imaging; NMDAR, N-methyl-D-aspartate receptor; OB, oligoclonal band.

## Treatment and Prognosis

There was no standard management for anti-AMPAR encephalitis. Therapies were usually chosen with reference to other autoimmune encephalitis (47), such as anti-NMDAR encephalitis. About 60-80% of autoimmune encephalitis with antibodies against neuronal surface antigens responded well to immunotherapy (48, 49). Similar to other neuronal surface autoantibody-mediated encephalitis, immunotherapy was recommended as early as possible after diagnosis (2, 50, 51).

First-line treatments included high-dose corticosteroids (methylprednisolone 1000 mg intravenously for 3 to 5 days), intravenous immunoglobulin (0.4 g/kg/day for 5 days) and plasma exchange. High-dose corticosteroids subsequently were followed by a tapering schedule. However, the optimal duration of steroid treatment remains inconclusive. There were 17 patients starting second-line treatment after first-line drugs. Timely initiation of second-line treatment was critical for patients non-reactive to first-line treatment. The exact timing of initiation of second-line therapy was unknown and may depend on the patients’ acceptance to side effects of first-line treatments, drug availability and neurologist’s preference. Major second-line drugs included rituximab and cyclophosphamide. Due to the small number of cases, the impact of second-line prevention on prognosis remained unclear. It was reported that patients who received second-line treatments during the first episode had a lower relapse rate and death rate than those who did not receive second-line immunotherapies (13, 48). Eight patients received long-term treatment in the remission period to prevent recurrence. Long-term treatment included azathioprine and mycophenolate mofetil. However, whether long-term treatment was needed for relapse prevention were still unclear.

In addition, symptomatic treatments were needed for mental disorders and seizures associated with anti-AMPAR-encephalitis. The effect of symptomatic treatment alone may be limited, and combined immunotherapy is usually required.

There were no significant differences in prognosis between patients with and without tumors in anti-AMPAR-encephalitis (13). Nevertheless, early treatment of tumors was important for a good prognosis (52). In 40 patients with concomitant tumors, 27 cases were treated for the neoplasms, such as tumor resection, chemotherapy and radiotherapy. Tumor screening should be conducted regularly for at least 2 years for anti-AMPAR encephalitis patients, even after resolution of neurological deficit (49, 53).

**Figure 3:**
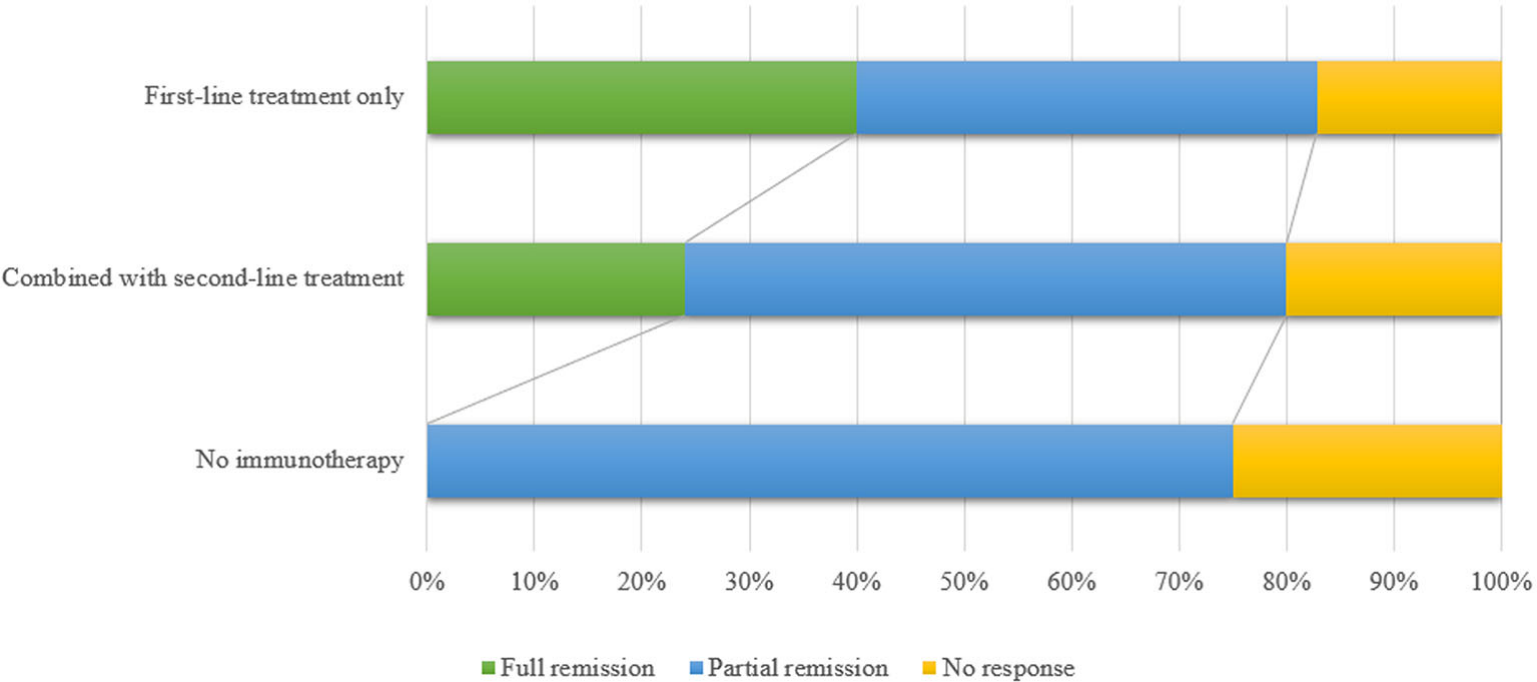
The figure shows the comparison of the efficacy between first-line immunotherapy only, first-line immunotherapy combined with second-line immunotherapy and no immunotherapy. Patients without immunotherapy had a relatively poor prognosis. First-line treatments included high-dose steroid pulse therapy, intravenous immunoglobulin and plasma exchange. Main second-line immunotherapy drugs included rituximab, cyclophosphamide and azathioprine. Full remission means the patients returned to baseline; partial remission means the patients showed partial recovery and partial sequelae; no response means patients did not respond to treatment medication.

Most anti-AMPAR encephalitis patients were responsive to immunotherapy. However, 18.5% patients (12/65, prognosis of 1 patient was not available) had a poor response. There were 30.8% patients (20/65) who returned to baseline. 50.8% patients (33/65) with anti-AMPAR encephalitis left some sequelae such as cognitive impairment and mental disorder (**Figure 3**). Previous study reported that the relapse rate was about 23.8% (48), which was higher in those who did not receive aggressive therapy (chemotherapy or rituximab) (13). The relapses of encephalitis did not mean the recurrence of tumors (6). Death rate was 16.7% (11/66), which was mostly related to the progression of the primary tumor. In a few cases, the causes of death were status epilepticus, cardiorespiratory arrest, myocardial infarction and urinary sepsis (6, 11).

## Conclusion

Anti-AMPAR encephalitis is an autoimmune disease of the central nervous system. Given the small number of reported cases, our understanding of anti-AMPAR encephalitis is still limited. Consensus was not reached regarding its diagnosis and standard management strategies. For patients with cognitive-psychiatric disorders with an acute or subacute onset, anti-AMPAR encephalitis should be considered. Serum and CSF AMPAR antibody should be tested as soon as possible for further confirmation. Timely immunotherapy should also be initiated upon diagnosis. An extensive screening for tumors, especially thymoma and lung cancer, is warranted in such patients.

## Author Contributions

T-YZ and M-TC drafted the initial manuscript, summarized available data and selected the references. YZ, Q-LL, and C-HS contributed to the manuscript. Y-XZ and SQ designed and revised the manuscript. All authors approved the final version of the manuscript.

## Funding

This study was supported by the Science and Technology Program of Zhejiang Province (2018C37132).

## Conflict of Interest

The authors declare that the research was conducted in the absence of any commercial or financial relationships that could be construed as a potential conflict of interest.
