# Supplementary material for: Anti-Alpha-Amino-3-Hydroxy-5-Methyl-4-Isoxazolepropionic Acid Receptor Encephalitis: A Review
Source: Front Immunol. 2021 May 21;12:652820. doi: 10.3389/fimmu.2021.652820 (PMC8175895; doi:10.3389/fimmu.2021.652820)
Supplement: Supplementary file 1 [file DataSheet_1.pdf]

Supplementary Figure

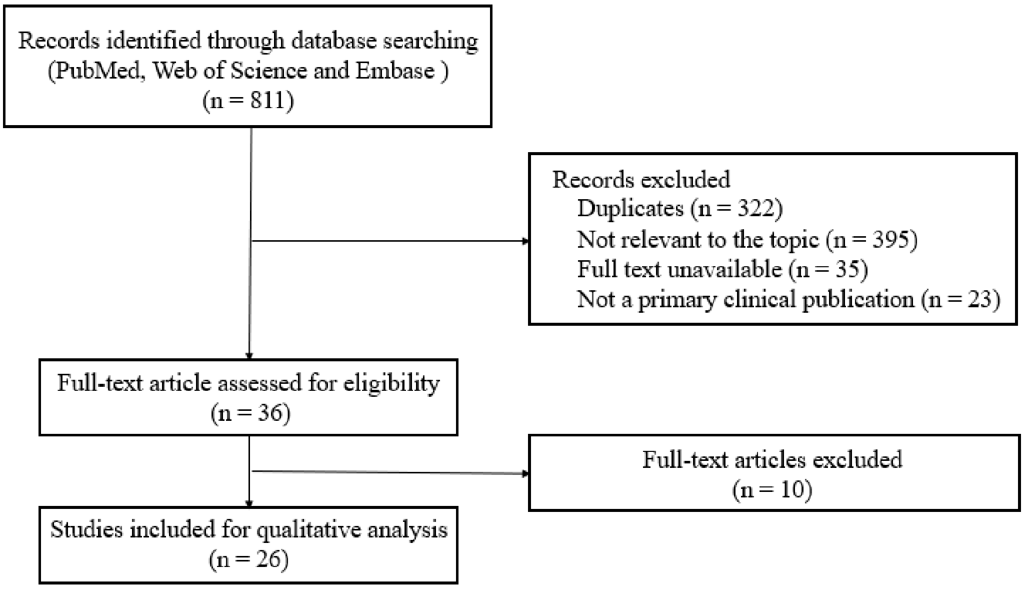

Supplementary Figure 1 Flowchart visualizes the selection process for included articles.

We used the search terms ((AMPA OR AMPAR OR anti-AMPA OR anti-AMPAR OR AMPAR-antibody OR AMPA receptor OR anti-alpha-amino-3-hydroxy-5-methyl-4-isoxazolepropionic acid) AND (encephalitis OR autoimmune encephalitis OR limbic encephalitis)) in PubMed, Web of Science and Embase.

Supplementary Table

Supplementary Table 1 Details of the clinical presentations, diagnostic tests and treatments (6-31)

Reference	No.	sex/ Age, y	Tumor	Additional antibodies	*Disease course	Symptom Presentation	Initial Brain MRI (T2/FLAIR) increased signal	EEG	Initial CSF	Treatment		#Treatment response
										Treatment of tumor	Immunotherapy	
Lai, 2009 (6)	1	F/65	-	-	Acute-subacute	Cognitive impairment, consciousness change, psychiatric disorder	Medial temporal lobes	Normal	30 WBC, 97 mg/dL protein, positive OB and ISAb	-	Plasma exchange, steroids, IVIg, azathioprine	Partial
	2	F/44	Thymic carcinoma	-	Acute-subacute	Cognitive impairment, consciousne	Normal	Diffuse theta activity;	44 WBC, 91 mg/dL protein, no	Tumor resection	Plasma exchange, steroids,	Partial

						ss change, psychiatric disorder, seizure		episodes of epileptic activity in left temporal lobe	OB, positive ISAb		IVIg, azathioprine	
	3	M/38	Malignant thymoma	GAD	Acute-subacute	Cognitive impairment, consciousness change, psychiatric disorder, seizure	Right medial and lateral temporal lobe, right frontal, left insular and left occipital regions	NA	7 WBC, 50 mg/dL protein, positive OB and ISAb	Tumor resection, radiation therapy	Steroids, plasma exchange, IVIg	Partial

	4	F/64	Non-SCLC		Acute-subacute	Seizures, consciousness change, cognitive impairment	Left medial temporal lobe	Slow activity in the right temporal region; no epileptic activity	75 WBC, 79 mg/dL protein	Tumor resection	Steroids	Full
	5	F/44	Thymoma	CV2/CRMP5	Acute-subacute	Consciousness change, psychiatric disorder, dyskinesia	NA, CT normal	NA	15 WBC, normal protein	Untreated	Unexpected dead	No
	6	F/38	-	-	Acute-subacute	Cognitive impairment, psychiatric disorder	Medial temporal lobes, left septal nucleus,	Normal	6 WBC, normal protein	-	IVIg, steroids	Full

							left cerebellum					
	7	F/87	-	-	Acute-subacute	Cognitive impairment, seizure	Medial temporal lobes; mild transient contrast enhancement in the left hippocampus	Diffuse slow activity (7-8c/sec), delta activity in anterior frontotemporal areas	Normal	-	Steroids	Partial
	8	F/61	Breast cancer		Acute-subacute	Cognitive impairment, consciousness change	Normal	Theta activity in posterior temporal regions	24 WBC, 420 mg/dL protein	Tumor resection	Steroids, plasma exchange	Partial

	9	F/59	SCLC	VGCC and SOX1	Acute-subacute	Cognitive impairment, psychiatric disorder, dyskinesia	Medial temporal lobes and medial orbitofrontal region	Bilateral sharp waves in temporal lobes; no seizures	17 WBC, 51 mg/dL protein, positive OB	Tumor resection, chemotherapy	Steroids, IVIg	Full
	10	F/67	Breast cancer	-	Chronic	Consciousness change, psychiatric disorder, cognitive impairment, insomnia	Temporal lobe	Sharp waves in temporal lobes	32 WBC, normal protein, no OBs	Tumor resection, radiation therapy	Steroids chemotherapy (including cyclophosphamide), IVIg	Partial

Bataller, 2010 (7)	11	F/67	Right breast ductal infiltrating adenocarcinoma (T1, N1, M0)	-	Acute	Psychiatric disorder, cognitive impairment, consciousness change, aphasis	Bilateral medial temporal lobe	Transient bilateral temporal sharp waves without clinical seizures	32 WBC (90% lymphocytes), normal protein and glucose levels, absent OB, negative cytology for neoplastic cells	Chemotherapy (Adriamycin)	IVIg, followed by cyclophosphamide	Partial

Graus, 2010 (8)	12	F/60	Malignant thymoma treated with radiotherapy and chemotherapy 6 years earlier without evidence of relapse	-	Acute	Consciousness change, psychiatric disorder	Normal	Diffuse slowing with occasional sharp waves over the frontal regions	Normal	Untreated	Steroids	Full

	13	F/58	-	-	Acute	Consciousness change, psychiatric disorder, aphasis	Normal	A normal background rhythm with episodic slow waves in frontal lobes	Normal	-	Steroids	Partial
Wei, 2013 (9)	14	F/30	-	VGKC/GAD	Acute	Psychiatric disorder, cognitive impairment, dyskinesia, consciousness change, seizure	Bilateral insula, medial temporal lobe and caudate nucleus	NA	70 WBC, 112 mg/dL protein, 49 mg/dLglucose	-	Steroids, plasma exchange, azathioprine	Partial

Spatola, 2014 (10)	15	F/33	-	-	Acute	Seizure, psychiatric disorder, cognitive impairment, dyskinesia	Left hippocampus	Bi-temporal regions with periodic lateralized epileptiform discharges (PLEDs) of shifting lateralization	9 WBC, normal protein and glucose, no OB	-	Steroids, plasma exchange, cyclophosphamide, rituximab	Full
Joubert, 2015 (11)	16	M/58	-	-	Acute	Consciousness change, cognitive impairment,	Bilateral hippocampi	Normal	Normal	-	Steroids, IVIg, cyclophosphamide	Partial

						aphasis						
	17	M/74	Hyper metabolic pulmonary lesion	Hu	Acute-subacute	Cognitive impairment, consciousness change, psychiatric disorder, insomnia	Bilateral hippocampi	Normal	38 WBC, 56 mg/dL protein, positive OB	Chemotherapy (carboplatin and VP16)	IVIg, plasma exchange	Partial
	18	F/56	-	-	Acute	Psychiatric disorder, cognitive impairment, consciousness change, dyskinesia	Bilateral hippocampi and gyri recti, right precentral area	Normal	220 WBC, normal protein	-	IVIg, steroids, rituximab	Partial
	19	F/43	-	LGI1,	Acute	Seizure,	Bilateral	Right	Normal	-	Steroids,	Full

				GAD65, IA2		cognitive impairment, dyskinesia, insomnia	hippocampi	temporal lobe seizures			IVIg	
	20	M/21	Thymic carcinoma with pleural extension and mediastinal lymph node metastasis	-	Acute-subacute	Consciousness change, psychiatric disorder, cognitive impairment, dyskinesia	Bilateral corpus striatum	Bifrontal sharp and slow waves without organized seizures	28 WBC, 70 mg/dL protein, no OB	Tumor resection, anticancerous chemotherapy and radiation therapy	Steroids and IVIg	Partial

	21	F/22	-	-	Acute	Consciousness change, psychiatric disorder, cognitive impairment, insomnia, dyskinesia	Left temporomesial areas, cortico-subcortical left temporal, insular, bilateral parietal lobes, right caudate nucleus and cerebellum	Normal	29 WBC, normal protein, no OB	-	Steroids, IVIg, cyclophosphamide, plasma exchange and rituximab	No
	22	F/92	-	-	Acute	Cognitive impairment,	Mild and diffuse	Normal	Normal WBC and	-	IVIg	Full

						dyskinesia	atrophy		protein, one OB			
Li, 2015 (12)	23	F/47	Type B1 thymoma	-	Acute	Psychiatric disorder, aphasis, consciousness change, dysarthria	Normal	Normal	Normal	Untreated	Steroids, azathioprine	Partial
Höftberger, 2015 (13)	24	F/42	Breast cancer	-	Subacute	Cognitive impairment, consciousness change, psychiatric disorder	Bilateral temporal lobe	Epileptiform activity	Normal	Tumor resection, chemotherapy	IVIg	Partial
	25	F/51	SCLC	-	Acute	Cognitive impairment, consciousne	Left medial temporal	NA	Normal	Chemotherapy, radiothera	Steroids	Full

						ss change, psychiatric disorder	lobe			py		
	26	M/59	SCLC	-	Chronic	Cognitive impairment, consciousness change, psychiatric disorder	Bilateral temporal lobes	Normal	6 WBC, 50 mg/dL protein	Chemotherapy, radiotherapy	IVIg	Full
	27	M/62	Malignant thymoma	-	Subacute	Cognitive impairment, consciousness change, psychiatric disorder	Bilateral temporal lobe and insula	NA	Normal	Tumor resection	Steroids, IVIg	Full
	28	F/63	SCLC	GABA(B)R, SOX1	Subacute	Cognitive impairment,	Medial temporal	NA	Normal WBC, 425	Chemotherapy,	-	Partial

						consciousness change, psychiatric disorder, seizure	lobes		mg/dL protein	radiotherapy		
	29	F/70	SCLC	-	Subacute	Cognitive impairment, consciousness change, psychiatric disorder	Normal	Epileptiform activity	Normal WBC, 64 mg/dL protein	Chemotherapy, radiotherapy	Steroids	Partial
	30	F/81	SCLC	SOX1	Subacute	Cognitive impairment, consciousness change, psychiatric disorder	Bilateral temporal lobe	Focal spike waves; polymorphic delta left frontotem	Normal	Chemotherapy	Steroids	No

								poral				
	31	F/33	-	-	Subacute	Cognitive impairment, consciousness change, psychiatric disorder, seizure	Bilateral hippocampi and amygdalae	Epileptiform activity	14 WBC, normal protein	-	Steroids, plasma exchange, rituximab, cyclophosphamide	No
	32	M/35	-	-	Acute	Cognitive impairment, consciousness change, psychiatric disorder	Medial temporal lobes	Focal activity temporal	23 WBC, proteins NA	-	Steroids, IVIg, rituximab	Partial
	33	F/64	-	-	Chronic	Cognitive impairment,	Left medial	Diffuse slowing	5 WBC, normal	-	Steroids, IVIg, plasma	Partial

						consciousness change, psychiatric disorder	temporal lobe	and focal abnormalities	protein		exchange	
	34	F/72	-	-	Chronic	Cognitive impairment, consciousness change, psychiatric disorder	Medial temporal lobes	General slowing	50 WBC, 49 mg/dL protein	-	Steroids, IVIg	Partial
	35	F/72	-	-	Chronic	Cognitive impairment, consciousness change, psychiatric disorder	Bilateral temporal lobe	Slow (theta) activity	52 WBC, 100 mg/dL protein	-	Steroids	Partial

	36	M/23	Thymoma	-	Acute	Cognitive impairment, psychiatric disorder, seizure	Basal ganglia	General slowing and epileptiform activity left temporal lobe	23 WBC, normal protein	Tumor resection	IVIg, steroids, rituximab	Partial
	37	F/25	Ovarian teratoma	NMDAR	Acute	Psychiatric disorder, consciousness change, seizure, dyskinesia, aphasis	Normal	NA	Normal	Tumor resection	Steroids, IVIg	Full
	38	F/53	Malignant	CRMP5	Subacute	Consciousness change,	Medial temporal	NA	164 WBC, 92 mg/dL	Tumor resection,	Steroids, IVIg	No

			thymoma			psychiatric disorder, seizure, autonomic dysfunction	lobes, frontobasal and caudate		protein	chemotherapy, radiotherapy		
	39	F/65	Breast cancer	-	Chronic	Cognitive impairment, consciousness change, psychiatric disorder, dyskinesia, seizure	Corpus callosum	Normal	Normal WBC, 71 mg/dL protein	Tumor resection, chemotherapy, radiotherapy	Steroids, IVIg, plasma exchange	Partial
	40	M/71	Thymic carcinoid	NMDAR	Subacute	Cognitive impairment, consciousness change,	Abnormality in the hypothalamic region	Generalized slowing	Normal WBC, elevated protein	Tumor resection	Steroids, plasma exchange	Full

						seizures, dyskinesia	with mass effect on pituitary gland; T2/ FLAIR increased signal in the right temporal lobe					
	41	M/72	Lung tumor	Amphi	Subacute	Cognitive impairment, dyskinesia, insomnia, psychiatric disorder	Normal	Normal	Normal WBC, 65 mg/dL protein	Untreated	Steroids	No

	42	M/62	-	CRMP5	Chronic	Cognitive impairment, consciousness change, psychiatric disorder, insomnia, dyskinesia	Basal ganglia	Focal activity	33 WBC, 173 mg/dL protein	-	Steroids, rituximab	No
	43	M/69	-	-	Chronic	Seizure, cognitive impairment, consciousness change, psychiatric disorder, aphasis, dyskinesia	Medial temporal lobe, cortical parietal lobe, cingulum, frontal lobe	Lateralized periodic slowing temporal and hippocampi	Normal	-	IVIg, plasma exchange	Partial

	44	F/29	Ovarian teratoma	-	Subacute	Cognitive impairment, consciousness change, psychiatric disorder, dysarthria	Insula, putamen and thalamus	Generalized slowing of background, focal slowing in right hemisphere	13 WBC, normal protein	Tumor resection	Steroids, IVIg, plasma exchange, rituximab	No
	45	F/38	-	-	Subacute	Psychiatric disorder, autonomic dysfunction	Normal	Normal	90 WBC, protein NA	-	NA	NA
Elamin, 2015 (14)	46	F/73	-	VGKC	Acute	Consciousness change, psychiatric disorder,	Normal	Severe bihemispheric slowing	Normal	-	IVIg, mycophenolate mofetil	Partial

						aphasis						
Dogan Onugoren, 2015 (15)	47	M/61	-	-	NA	Cognitive impairment	Bilateral mediotemporal	Normal	Normal WBC, proteins NA, no ISAb	-	Steroids, azathioprine	Partial
	48	M/62	-	-	NA	Cognitive impairment, psychiatric disorder	Bilateral mediotemporal	Normal	16 WBC, proteins NA, no ISAb	-	Steroids, rituximab, IVIg, plasma exchange	No
	49	F/61	Ovarian cancer	-	NA	Cognitive impairment, psychiatric disorder	Bilateral mediotemporal	Normal	Normal WBC, proteins NA, no ISAb	Chemotherapy	Steroids, mycophenolate mofetil, IVIg, plasma exchange	Partial

Quaranta, 2015 (16)	50	F/14	-	-	Acute	Psychiatric disorder, aphasis, cognitive impairment, consciousness change, dyskinesia	Normal	Normal	NA	-	-	Partial
Boangher, 2016 (17)	51	F/66	Situ bladder carcinoma, SCLC	NMDAR	NA	Consciousness change, cognitive impairment, psychiatric disorder	Normal	Normal	18 WBC, normal protein and glucose	Tumor resection (situ bladder carcinoma), chemotherapy (SCLC)	Steroids, plasma exchange, Mycophenolate mofetil	Partial

Yang, 2016 (18)	52	M/40	Invasive thymoma, multiple pulmonary metastases	CV2/CRMP5	Acute	Seizure, cognitive impairment, consciousness change, dyskinesia, autonomic dysfunction	Right temporal and parietal cortex	Normal	52 WBC, 63.2 mg/dL protein, normal glucose, no OB	Untreated	IVIg	No
Zhu, 2017 (19)	53	F/54	-	-	Acute	Cognitive impairment, consciousness change	Left temporal lobe and hippocampus	Paroxysmal delta waves in the left temporal lobe	63 WBC, 50 mg/dL protein	-	IVIG, steroids	Partial

Koh, 2018 (20)	54	M/19	Ewing sarcoma, multiple pulmonary metastases	-	Acute	Consciousness change, cognitive impairment	Bilateral hippocampi	NA	lymphocytosis with a raised protein level	Untreated	IVIg	No
Omi, 2018 (21)	55	F/34	Thymoma, type B3	-	Acute	Consciousness change, psychiatric disorder, dyskinesia, cognitive impairment, seizure	Lower region of the left caudate nucleus	Focal epileptic discharges at the left temporal-parietal region	Normal	Tumor resection	Steroids	Full

Zhu, 2018 (22)	56	M/51	SCLC	-	Subacute	Cognitive impairment, psychiatric disorder	Normal	Normal	Normal	Tumor resection	IVIg,	Partial
Samad, 2018 (23)	57	F/69	Medullary thyroid cancer	-	Acute	Cognitive impairment, consciousness change, psychiatric disorder	Normal	NA	WBC NA, 0.62 mmol/L proteins, 3.7 mmol/L glucose	Untreated	IVIg	Full
Laurido-Soto, 2019	58	M/44	Thymoma	CRMP5	NA	Cognitive impairment, psychiatric disorder, dyskinesia	Bilateral hippocampi	Generalized slowing	lymphocytic pleocytosis	Untreated	Steroids, IVIg, a single dose of rituximab	Full

(24)	59	M/18	-	-	Subacute	Cognitive impairment, psychiatric disorder, consciousness change, dyskinesia	Bilateral cerebellar hemispheres	NA	lymphocytic pleocytosis, normal protein and glucose	-	IVIg, rituximab, steroids	Full
Urriola, 2019 (25)	60	F/44	Thymoma	-	Acute	Consciousness change, psychiatric disorder	Medial temporal lobes	Isoelectric pattern	pleocytosis, normal protein and glucose	Untreated	Plasma exchange, steroids, IVIg, rituximab, cyclophosphamide	Partial

Danesh mand, 2019 (26)	61	F/61	Malignant melanoma	-	Acute	Cognitive impairment, consciousness change, dyskinesia	Bilateral corpus striatum	Diffuse slowing with no evidence of epileptiform discharges	71 WBC, 63 mg/dL protein, normal glucose	Chemotherapy (a BRAF inhibitor [vemurafenib] and an MEK inhibitor [cobimetinib])	Steroids, IVIg, plasma exchange, rituximab	Full
Luo, 2019 (27)	62	F/50	Thymoma	-	Subacute	Cognitive impairment, psychiatric disorder, consciousness change, aphasis,	Bilateral medial temporal lobe and hippocampi	A wide range of abnormalities in 6 – 8 Hz low to middle slow	Normal	Untreated	Steroids, azathioprine	Partial

						dysarthria, autonomic dysfunction		waves				
Jia, 2020 (28)	63	M/26	Lymphoma	CV2/CRMP5	Acute	Insomnia, consciousness change, dyskinesia, psychiatric disorder, autonomic dysfunction	Bilateral cerebellar hemispheres, cerebellar vermis, left hippocampus, basal ganglia region and bilateral frontoparietal cortex	NA	11 WBC, normal biochemistry	Untreated	Steroids, IVIg	No

Wei, 2020 (29)	64	F/66	Left breast invasive ductal carcinoma	-	Acute	Dyskinesia, psychiatric disorder, aphasis, cognitive impairment, consciousness change	Normal	Normal	NA	Untreated	Steroids, plasma exchange	Full
Safadi, 2020 (30)	65	M/30	Thymoma, type B2	-	Acute	Seizures, dyskinesia, autonomic dysfunction	FLAIR hyperintensity scattered within mostly the subcortical white matter as	NA	97 WBC, 70 mg/dL protein, 65 mg/dl glucose	Untreated	IVIg, steroids, plasma exchange, cyclophosphamide	Full

							well as an enhancing lesion adjacent to the left caudate nucleus					
Qiao, 2020 (31)	66	M/32 months	-	-	Acute	Psychiatric disorder, seizures, dyskinesia, consciousness change	Normal	A widespread abnormality and detected a large number of spikes and multiple	Normal	-	Steroids, IVIg	Full

								spines-slow waves				

Abbreviations: Amphi: anti-amphiphysin antibody; CSF: cerebrospinal fluid; CT: computerized tomography; CV2/CRMP5: anti-collapsin response-mediator protein-5 antibody; EEG: electroencephalogram; F: female; FLAIR: fluid-attenuated inversion-recovery; GABA(B)R: anti-gamma-aminobutyric acid receptor type B; GAD: anti-glutamic acid decarboxylase antibody; IA2: anti-islet cell antigen 512 antibody; ISAb: intrathecal synthesis of antibodies; IVIg: intravenous immunoglobulin; LGI1: anti-leucine-rich glioma-inactivated 1; M: male; MRI: magnetic resonance imaging; NA: not available; NMDAR: anti-N-methyl-D-aspartate receptor antibody; OB: oligoclonal bands; SCLC: small cell lung cancer; SOX1: anti-Sry-like high mobility group box antibody; VGCC: voltage-gated calcium channels antibody; VGKC: anti-voltage-gated potassium channels antibody; VP16: etoposide.; WBC: white blood cell.

*Acute: progression of less than 3 weeks; Subacute: progression of less than 3 months; Chronic: progression of more than 3 months.

#Full: full remission; Partial: partial remission; No: no response.
